# Gentiopicroside Produces Endothelium-Independent Vasodilation by Deactivating the PI3K/Akt/Rho-Kinase Pathway in Isolated Rat Thoracic Aorta

**DOI:** 10.1155/2021/5565748

**Published:** 2021-05-14

**Authors:** Shangping Xing, Feifei Nong, Jialiang Qin, Huicai Huang, Ruoting Zhan, Weiwen Chen

**Affiliations:** ^1^Research Center of Chinese Herbal Resource Science and Engineering, Key Laboratory of Chinese Medicinal Resource from Lingnan, Ministry of Education, Guangzhou University of Chinese Medicine, Guangzhou 510006, China; ^2^Pi-Wei Institute, Science and Technology Innovation Center, Guangzhou University of Chinese Medicine, Guangzhou 510006, China

## Abstract

Gentiopicroside (GPS), a main active secoiridoid glucoside derived from the roots of perennial herbs in the *Gentianaceae* family, has antispasmodic and relaxant effects. However, the vasorelaxant effects of GPS on aortic rings and the molecular mechanisms involved in these effects are not yet clear. Therefore, we investigated whether GPS inhibits phenylephrine- (PE-) or KCl-induced contractions in isolated rat thoracic aortic rings. The present study found that GPS produced a dose-dependent relaxation in aortic rings precontracted with PE or KCl and significantly reduced CaCl_2_-, narciclasine- (Rho-kinase activator-), and phorbol-12,13-diacetate- (PKC activator-) induced vasocontractions. Pretreatment with NG-Nitroarginine methyl ester hydrochloride (L-NAME, NOS inhibitor), methylene blue (sGC inhibitor), indomethacin (COX inhibitor), 4-aminopyridine (K_V_ channel inhibitor), and glibenclamide (K_ATP_ channel inhibitor) had no influence on the vasorelaxant effect of GPS, while BaCl_2_ (K_ir_ channel inhibitor), tetraethylammonium chloride (K_Ca_ channel inhibitor), ruthenium red (RYR inhibitor), and heparin (IP_3_R inhibitor) significantly reduced GPS-induced vasorelaxation. Moreover, GPS pretreatment remarkably inhibited the influx of Ca^2+^ in vascular smooth muscle cells stimulated using KCl or PE-containing CaCl_2_ solution. Western blot analysis confirmed that GPS treatment inhibited PE-induced increases in the protein levels of p-Akt, p-myosin light chain (MLC), and p-myosin-binding subunit of myosin phosphatase 1 (MYPT1) in the aortic rings. Additionally, the vasorelaxation activity of GPS was attenuated upon pretreatment with LY294002 (PI3K/Akt inhibitor), Y27632 (Rho-kinase inhibitor), and verapamil (L-type Ca^2+^ channel inhibitor). These findings demonstrate that GPS exhibits endothelium-independent vasorelaxant effects through inhibition of voltage-dependent, receptor-operated, and inositol triphosphate receptor (IP_3_R)/ryanodine receptor- (RYR-) mediated Ca^2+^ channels as well as the PI3K/Akt/Rho-kinase signaling pathway.

## 1. Introduction

Hypertension, which is associated with vasoconstriction and vascular remodeling, is a serious threat to global public health by causing cardiovascular diseases, such as atherosclerosis, myocardial infarction, and vascular hypertrophy [1–4]. The incidence and mortality rates of hypertension are rapidly increasing worldwide; hypertension causes approximately 9 million deaths each year, and the total number of hypertensive patients in the world is expected to reach 1.5 billion by 2025 [5, 6]. Therefore, lowering blood pressure and relaxing blood vessels can greatly alleviate the risk of cardiovascular disease development caused by elevated blood pressure.

Accumulating evidence [7–9] suggests that the hypercontractility of vascular smooth muscle (VSM) is closely related to raised blood pressure, while intracellular calcium concentration ([Ca^2+^]_in_) is the primary regulator of tension in VSM. Ca^2+^ is a critical factor in excitation-contraction coupling in VSM, an increase in [Ca^2+^]_in_ results in vasoconstriction and a decrease in [Ca^2+^]_in_ results in vasodilation [10]. It is well known that the regulation of vascular tone is mainly triggered by releasing vasodilator factors [nitric oxide (NO), prostacyclin (PGI_2_)], changing the resting membrane potential (K^+^ channels), the influx of extracellular Ca^2+^ through receptor-operated calcium channel (ROCC) and voltage-dependent calcium channel (VDCC, including L-type Ca^2+^ channels), and the release of intracellular Ca^2+^ from sarcoplasmic reticulum [11, 12]. Additionally, many signaling pathways have been reported to play an essential role in vasoconstriction processes [13]. For example, PI3K/Akt upon activation can induce the VSM constriction by coupling membrane receptors to L-type Ca^2+^ channels [14, 15]. PKC and Rho-kinase Ca^2+^-sensitizing pathways leading to myosin phosphatase inhibition are critically involved in *α*1-adrenoceptor-mediated VSM contraction [16]. Although many vasodilators are commercially available such as nitroglycerin (NO donor drug), verapamil (Ca^2+^ channel antagonist), and fasudil (ROCK inhibitor), they are limited by their adverse effects and patient compliance [17, 18]. Therefore, the development of natural vasodilator compounds has far-reaching research significance and high relevance for the discovery of new treatment of cardiovascular diseases.

**Figure 1:**
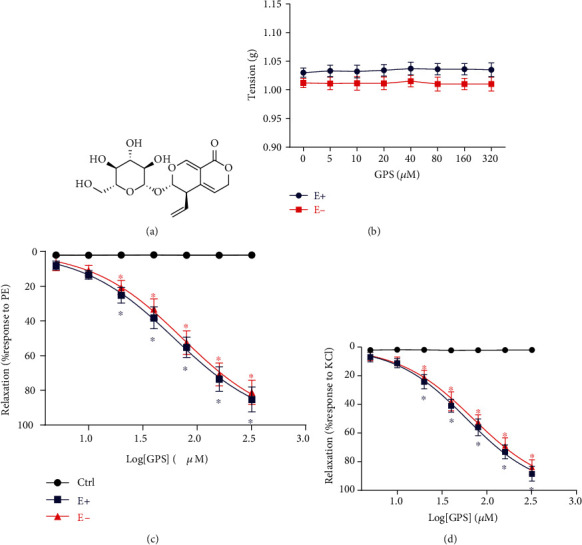
Effects of GPS on the vasorelaxation of thoracic aortic rings. (a) Chemical structure of GPS. (b) Direct effects of GPS (5, 10, 20, 40, 80, 160, and 320 *μΜ*) on the thoracic aorta tension. (c) Cumulative concentration-response curves of GPS (5, 10, 20, 40, 80, 160, and 320 *μΜ*) on endothelium-intact (E+) and endothelium-denuded (E-) aortic rings precontracted with 1 *μ*M PE or (d) 60 mM KCl. Data are presented as mean ± SEM (*n* = 6). ^∗^*p* < 0.05 compared to the control group.

Gentiopicroside (GPS, C_16_H_20_O_9_, Figure 1(a)) is a secoiridoid glucoside that is isolated from the roots of perennial herbs in the *Gentianaceae* family, such as *Gentiana straminea* Maxim., *Gentiana macrophylla* Pall., *Gentiana manshurica* Kitag., *Gentiana dahurica* Fisch., and *Gentiana scabra* Bge., which are used widely as medicinal herbs in China for the treatment of rheumatoid arthritis, hemiplegia, arthralgia, stroke, and hypertension [19]. GPS has been proven to display potential protective effects against osteoarthritis, hepatitis, diabetic renal fibrosis, osteoclastogenesis, and alcoholic hepatosteatosis [20]. Kesavan et al. [21] reported that *Gentiana lutea* root extracts consisting of GPS significantly inhibit the proliferation of VSMCs induced by platelet-derived growth factor-BB, which may have a cardiovascular protective effect in the prevention and treatment of atherosclerosis. Given that GPS can inhibit the spontaneous contractions of smooth muscle induced by histamine, KCl, and BaCl_2_ in isolated pig ileum [22], we hypothesized that GPS may inhibit vascular contraction by blocking ion channels or the corresponding signal transduction pathways.

To the best of our knowledge, the effects of GPS on aortic rings and the molecular mechanisms involved in these effects have not yet been clarified. Therefore, this study aims to investigate the effects of GPS on the vasoconstriction of aortic rings induced by PE and KCl. We also explored the underlying mechanisms of the GPS-promoted vasodilatation effects by studying Ca^2+^ and K^+^ channels and the PI3K/Akt/Rho-kinase signaling pathway. These data could provide a novel insight into the molecular mechanisms underlying the vasodilatory effects of GPS.

## 2. Materials and Methods

### 2.1. Preparation of Rat Thoracic Aortic Rings

Fifty-one specific pathogen-free grade healthy male Sprague-Dawley rats (4-6 months old and weighing an average 250 g) were obtained from the Experimental Animal Center of Guangzhou University of Chinese Medicine (no: SCXK-2013-0020). All procedures in this study were approved by the Ethics Committee for the Use of Experimental Animals of Guangzhou University of Chinese Medicine (Permit no: 20190513056). As described previously [23], after the SD rats were euthanized, the thoracic aorta was carefully dissected and placed into ice-cold modified Krebs solution. The isolated aortas were cleaned of adipose and connective tissue and cut into 3-4 mm long rings, which were then mounted with two stainless steel hooks into an organ bath containing Krebs solution (gassed with 95% O_2_ and 5% CO_2_ at 37°C) at an initial force of 1 g tension. The alternation of isometric tension was recorded with a force-displacement transducer connected to a ML870 Power Lab Biological Signal Collection System (AD Instruments, Castle Hill, NSW, Australia). The endothelium of the aortic ring was removed carefully by rotating a manipulator inside the ring lumen, and its absence was verified by examining the capacity of 10 *μ*M acetylcholine to induce less than 10% relaxation of rings precontracted with 1 *μ*M PE. The endothelium was considered intact when the relaxation was more than 80% in response to acetylcholine. Only the aortic rings that met this standard were used for the subsequent experiments.

### 2.2. Action of GPS on Baseline Tension

After the aortic rings, with or without endothelium, were stabilized at primary 1 g tension, cumulative concentrations of GPS (0, 5, 10, 20, 40, 80, 160, and 320 *μΜ*) were added to the organ chambers. Changes in vascular tension were recorded, and a cumulative concentration-response curve for GPS was obtained.

### 2.3. Effect of GPS on Aortic Rings Precontracted with PE and KCl

After equilibration for 60 min, PE (1 *μ*M) or KCl (60 mM) was used to induce a steady contraction in the aortic ring with or without endothelium. This was followed by the addition of cumulative concentrations of GPS (0, 5, 10, 20, 40, 80, 160, and 320 *μΜ*) to the ring for 20 min to verify its vasorelaxant activity. The vasodilation rate (%) was calculated as follows: Relaxation (%) = (maximal tension by PE or KCl − tension after incubation with corresponding compounds)/(maximal tension by PE or KCl − basal tension before precontraction with PE or KCl) × 100%.

### 2.4. Effects of Various Inhibitors on GPS-Induced Vasodilation

To elucidate the role of the endothelium, K^+^ channel, and PI3K/Akt/Rho-kinase pathways in GPS-mediated vasodilation, the aortic rings with intact endothelium were preincubated with nitric oxide synthase (NOS) inhibitor (100 *μ*M L-NAME), cyclooxygenase (COX) inhibitor (100 *μ*M indomethacin), and soluble guanylyl cyclase (sGC) inhibitor (100 *μ*M methylene blue), respectively, for 30 min, while the aortic rings without endothelium were preincubated with different K^+^ channel blockers of tetraethylammonium chloride (TEA, 10 mM), 4-aminopyridine (4-AP, 1 mM), BaCl_2_ (1 mM), glibenclamide (0.01 mM), L-type Ca^2+^ channel inhibitor (100 *μ*M verapamil), PI3K/Akt inhibitor (10 *μ*M LY294002), and Rho-kinase inhibitor (10 *μ*M Y27632) for 30 min (all inhibitors were obtained from MedChem Express, NJ, USA). After achieving a plateau of PE-induced contracted tension, GPS was added in a cumulative manner (20, 80, and 160 *μΜ*) for 20 min, and the concentration-response curves were recorded.

### 2.5. Effect of GPS on Extracellular Ca^2+^-Induced Contraction

After the aortic rings without endothelium were incubated with Ca^2+^-free Krebs solution [containing 0.5 mM Ethylenebis (oxyethylenenitrilo) tetraacetic acid (EGTA) and 60 mM KCl] for 30 min, the rings were treated with GPS (20, 80, and 160 *μΜ*) for 20 min, and subsequently, CaCl_2_ (0.1, 0.3, 1, 3, and 10 mM) was added cumulatively to obtain concentration-response curves.

### 2.6. Cell Culture

The rat thoracic aorta vascular smooth muscle A7r5 cell line was purchased from Shanghai Cell Bank (Shanghai, China). The cells were cultured in high-glucose Dulbecco's Modified Eagle Medium (DMEM) supplemented with 10% fetal bovine serum and 1% penicillin-streptomycin solution (all these reagents were obtained from Gibco, Grand Island, NY, USA) and incubated at 37°C with 5% CO_2_.

### 2.7. Effect of GPS on [Ca^2+^]_in_ in A7r5 Cells

A7r5 cells (5 × 10^4^ cells/well) in chamber slides were loaded with 10 *μΜ* Fluo-4/AM (Thermo Fisher Scientific, Grand Island, NY, USA) in Ca^2+^-free Krebs solution for 30 min at 37°C with 5% CO_2_ in the dark, as described previously [24]. A7r5 cells were then washed thrice with Ca^2+^-free Krebs solution and incubated with Ca^2+^-free Krebs solution for 20 min to generate free Fluo-4. After treatment with GPS (20, 80, and 160 *μΜ*) for 30 min at 37°C with 5% CO_2_ in the dark, 100 mM KCl in Krebs solution, or 1 *μ*M PE-2.5 mM CaCl_2_ in Ca^2+^-free Krebs solution was added to induce fluorescence emission during detection. Fluorescence was captured using laser confocal microscopy (Zeiss 880, Jena, Germany).

### 2.8. Effect of GPS on Intracellular Ca^2+^

Aortic rings without endothelium were firstly contracted with 60 mM KCl in normal Krebs solution to assure rich Ca^2+^ storage in the sarcoplasmic reticulum. Following this, the rings were allowed to rest in Ca^2+^-free Krebs solution (containing 0.5 mM EGTA) for 30 min. (1) On one hand, the rings were incubated with 10 *μ*M narciclasine (Rho-kinase activator) or 10 *μ*M phorbol-12,13-diacetate (PKC activator) and then treated with GPS (20, 80, and 160 *μΜ*) for 20 min. (2) On the other hand, the rings were incubated with 50 mg/L heparin [inositol triphosphate receptor (IP_3_R) inhibitor] or 10 *μ*M ruthenium red [ryanodine receptor (RYR) inhibitor] for 30 min (all activators and inhibitors were obtained from MedChem Express), and then, the rings precontracted with PE (1 *μ*M) were treated with GPS (20, 80, and 160 *μΜ*) for 20 min, followed by recording of the concentration-response curves.

### 2.9. Western Blot Analysis

Isolated aortic rings without endothelium were transferred to DMEM and incubated at 37°C with 5% CO_2_. PE was added to the medium for 30 min, followed by GPS (160 *μΜ*) for 20 min. Aortic rings were snap frozen with liquid nitrogen; then, the protein was extracted using radioimmunoprecipitation assay (RIPA) lysis buffer mixed with 1% phenylmethanesulfonyl fluoride (PMSF). According to the standard WB procedure, the membranes were incubated with primary antibodies against GAPDH, Akt, p-Akt, MLC, p-MLC, MYPT1, and p-MYPT1 (1 : 1000 dilution, Cell Signaling Technologies, Beverly, MA, USA) overnight at 4°C, followed by incubation with the corresponding secondary antibody for 1 h. The protein bands were visualized using an ECL reagent (EMD Millipore) and analyzed using the Tanon 5200 image acquisition system (Tanon Science and Technology Co., Ltd., Shanghai, China).

### 2.10. Statistical Analysis

All data are expressed as mean ± SEM. Data were plotted using the GraphPad Prism software (version 6.0; GraphPad Software, Inc.), with sigmoidal curve fitting performed by nonlinear regression using the Prism software. The maximal relaxation or contraction response was presented as *E*_max_, and the half-maximal effective concentration was presented as EC_50_. Data were analyzed by one-way and two-way analysis of variance (ANOVA), followed by Bonferroni's as a posttest, using the SPSS software (version 23.0; IBM Corp.). Differences with *p* < 0.05 were considered statistically significant.

## 3. Results

### 3.1. Effects of GPS on PE- or KCl-Induced Contractions in Aortic Rings

As shown in Figure 1(b), there was no change in the tension of rat thoracic aortic rings with or without endothelium after direct application of cumulative concentrations of GPS (5-320 *μΜ*). The results indicated that GPS has no direct vasoconstriction and vasorelaxation effects on aortic rings that sustained resting tension. However, GPS (5-320 *μΜ*) produced a dose-dependent relaxation in the aortic rings with or without endothelium that were precontracted with PE or KCl (Figures 1(c) and 1(d)). The half-maximal effective concentration (EC_50_) values of the relaxant effects of GPS for endothelium-intact and endothelium-denuded aortic rings were 76.56 ± 3.62 and 78.81 ± 3.06 *μΜ*, respectively, in case of PE-induced contraction, and 72.68 ± 4.18 and 75.19 ± 4.22 *μΜ*, respectively, in case of KCl-induced contraction. No significant difference was observed in GPS relaxing PE- or KCl-induced contractions between endothelium-intact and endothelium-denuded aortic rings (*E*_max_/PE: E+, 85.29 ± 3.55% vs. E-, 81.32 ± 3.46%, *p* > 0.05; *E*_max_/KCl: E+, 88.52 ± 2.88% vs. E-, 83.89 ± 2.69%, *p* > 0.05). These results showed that the vasodilation of GPS on aortic rings precontracted with PE or KCl was endothelium-independent.

### 3.2. Effects of Endothelial Dilated Mediators on GPS-Induced Vasorelaxation

**Figure 2:**
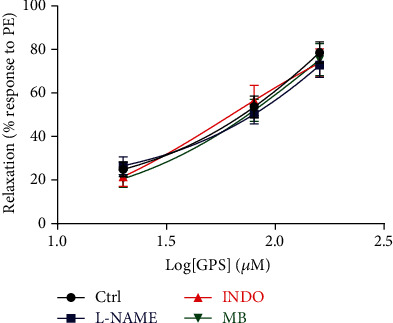
Effects of dilated mediators, including L-NAME, indomethacin (INDO), and methylene blue (MB) on GPS- (20, 80, and 160 *μ*M) induced vasorelaxation in endothelium-intact aortic rings precontracted with PE. Data are presented as mean ± SEM (*n* = 6). ^∗^*p* < 0.05 compared to the control group.

It has been reported [25] that NOS, sGC, and COX are essential in the formation and activation of NO and PGI_2_, which are the main endothelium-dependent relaxing factors. Pretreatment with L-NAME (NOS inhibitor), indomethacin (COX inhibitor), and methylene blue (sGC inhibitor) had no interfering effect on GPS- (20, 80, and 160 *μ*M) induced vasorelaxation (*E*_max_: 72.76 ± 2.66%, 73.80 ± 2.71%, 75.28 ± 3.19%) in aortic rings precontracted with PE when compared to control group (*E*_max_: 78.58 ± 2.13%) (*p* > 0.05), which further confirmed that GPS-induced vasorelaxation was endothelium-independent (Figure 2).

### 3.3. Effects of K^+^ Channels on GPS-Induced Vasorelaxation

**Figure 3:**
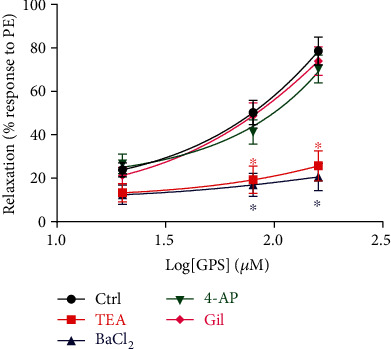
Effects of K^+^ channel blockers, including tetraethylammonium chloride (TEA), BaCl_2_, 4-aminopyridine (4-AP), and glibenclamide (Gli) on GPS- (20, 80, and 160 *μ*M) induced vasorelaxation in endothelium-denuded aortic rings precontracted with PE. Data are presented as mean ± SEM (*n* = 6). ^∗^*p* < 0.05 compared to the control group.

As shown in Figure 3, pretreatment with Gli [ATP-sensitive K^+^ (K_ATP_) channel inhibitor] and 4-AP [voltage-dependent K^+^ (K_V_) channel inhibitor] had no effect on GPS- (20, 80, and 160 *μ*M) induced vasorelaxation (*E*_max_: 73.98 ± 3.17%, 70.29 ± 2.92%) in aortic rings without endothelium that were precontracted with PE, compared to the control group (*E*_max_: 77.56 ± 3.03%) (*p* > 0.05). In contrast, pretreatment with TEA [Ca^2+^-activated K^+^ (K_Ca_) channel inhibitor] or BaCl_2_ [inward rectifier K^+^ (K_ir_) channel inhibitor] significantly reduced the vasorelaxation by GPS with *E*_max_ of 25.68 ± 3.71% or 20.58 ± 3.35%, compared to the control group (*p* < 0.05). These results indicated that the activation of K^+^ channels may be related to the vasodilation of GPS.

### 3.4. GPS Inhibited Extracellular Ca^2+^ Influx in Aortic Rings and A7r5 Cells

**Figure 4:**
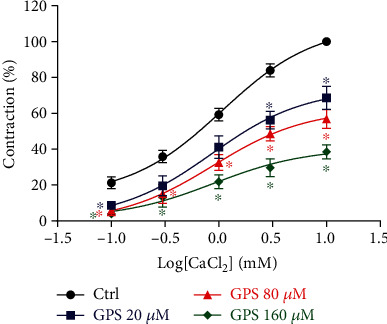
Effects of GPS (20, 80, and 160 *μ*M) on CaCl_2_- (0.1, 0.3, 1, 3, and 10 mM) induced contractions of endothelium-denuded aortic rings in high K^+^ and Ca^2+^-free Krebs solution. Data are presented as mean ± SEM (*n* = 6). ^∗^*p* < 0.05 compared to the control group.

**Figure 5:**
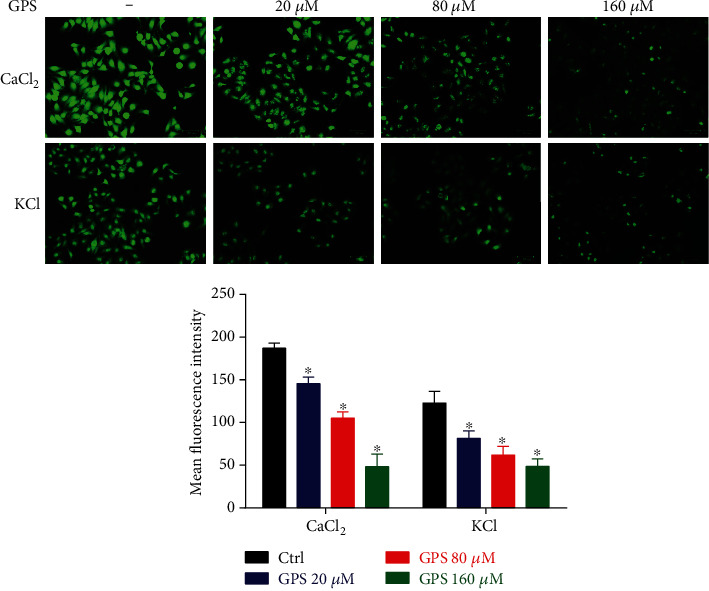
Effects of GPS (20, 80, and 160 *μ*M) on the fluorescence intensity of vascular smooth muscle A7r5 cells stimulated using 1 *μ*M PE-2.5 mM CaCl_2_ in Ca^2+^-free Krebs solution or 100 mM KCl in Krebs solution. Images were taken using laser confocal microscopy. Scale bar: 50 *μ*m. Green fluorescence intensities represent the intracellular calcium concentration ([Ca^2+^]_in_) levels. Data are presented as mean ± SEM (*n* = 6). ^∗^*p* < 0.05 compared to the control group.

In the high K^+^ and Ca^2+^-free Krebs solutions, the cumulative addition of CaCl_2_ (0.1, 0.3, 1, 3, and 10 mM) induced concentration-dependent contractions in the aortic rings without endothelium, while GPS (20, 80, and 160 *μ*M) treatment significantly inhibited these CaCl_2_-induced contractions (*E*_max_: 68.65 ± 3.89%, 56.92 ± 3.52%, 38.52 ± 2.23%, vs. 100 ± 0.01% in control group, *p* < 0.05) (Figure 4). To further investigate the effect of GPS on extracellular Ca^2+^ influx, we used a Fluo-4/AM molecular probe to observe intracellular Ca^2+^ fluorescence intensity in rat VSM A7r5 cells. The results revealed that pretreatment with GPS (20, 80, and 160 *μ*M) concentration dependently and significantly reduced the fluorescence intensity of A7r5 cells stimulated with CaCl_2_ or KCl (Figure 5). These results confirmed that the inhibition of extracellular Ca^2+^ influx might be involved in the underlying mechanism of GPS in the promotion of vasorelaxation.

### 3.5. GPS Inhibited Intracellular Ca^2+^ Release in Aortic Rings

**Figure 6:**
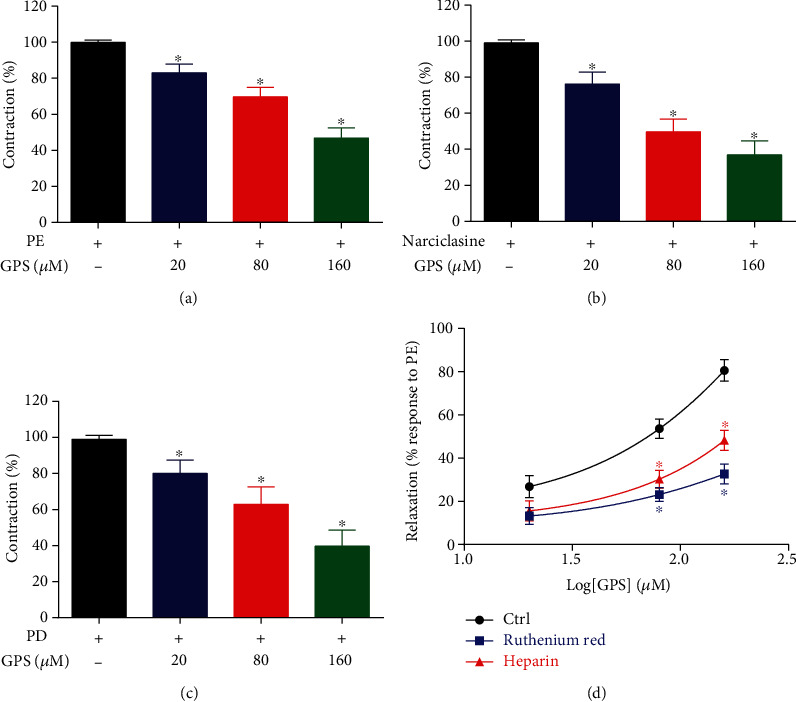
Effects of GPS treatment on intracellular Ca^2+^ release. (a) Endothelium-denuded aortic rings were precontracted with PE, (b) narciclasine, and (c) phorbol-12,13-diacetate (PD), followed by GPS (20, 80, and 160 *μ*M) treatment and detection of the vasorelaxation of the rings in Ca^2+^-free Krebs solution. (d) Endothelium-denuded aortic rings were incubated with ruthenium red and heparin in Ca^2+^-free Krebs solution, and then, the rings were treated with GPS (20, 80, and 160 *μΜ*) after precontracted with PE. Data are presented as mean ± SEM (*n* = 6). ^∗^*p* < 0.05 compared to the control group.

Preincubation with GPS (20, 80, and 160 *μ*M) significantly decreased PE-, narciclasine- (Rho-kinase activator) or phorbol-12,13-diacetate- (PKC activator) induced contractions of aortic rings in Ca^2+^-free solution, compared to the control group (*p* < 0.05, Figures 6(a)–6(c)). The results indicated that GPS could inhibit the vasoconstriction caused by the release of intracellular Ca^2+^. Furthermore, pretreatment with ruthenium red (RYR antagonist) or heparin (IP_3_R antagonist) significantly reduced the vasodilatory effects of GPS on aortic rings precontracted with PE in Ca^2+^-free solution (*E*_max_: 32.66 ± 2.91% in ruthenium red, 48.25 ± 3.22% in heparin vs. 80.58 ± 3.37% in control group, *p* < 0.05 in both, respectively) (Figure 6(d)). These results suggested that the mechanisms by which GPS inhibits vasoconstriction seem to be associated with the blockade of IP_3_R/RYR-mediated intracellular Ca^2+^ channels in the sarcoplasmic reticulum and the Rho-kinase-PKC induced MLC phosphorylation.

### 3.6. GPS Suppressed the Activation of the PI3K/Akt/Rho-Kinase Signaling Pathway

**Figure 7:**
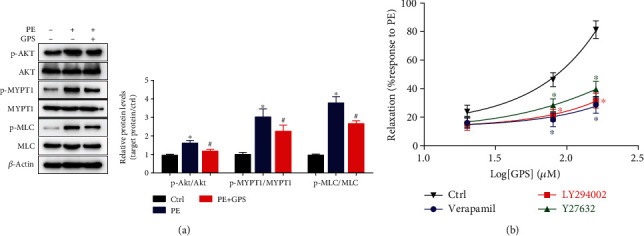
Effects of GPS on the PI3K/Akt/Rho-kinase pathway. (a) Protein expression levels of p-Akt, Akt, p-MYPT1, MYPT1, p-MLC, and MLC in aortic rings without endothelium were analyzed using western blotting. Data are presented as SEM (*n* = 3). ^∗^*p* < 0.05 compared to the control group; ^#^*p* < 0.05 compared to the PE group. (b) Endothelium-denuded aortic rings were preincubated with verapamil, LY294002, and Y27632, and then, the rings were treated with GPS (20, 80, and 160 *μΜ*) after precontracted with PE. Data are presented as mean ± SEM (*n* = 6). ^∗^*p* < 0.05 compared to the control group.

Increasing evidence suggests that the PI3K/Akt/Rho-kinase signaling pathway plays a crucial role in vasoconstriction by stimulating the L-type Ca^2+^ channel [26]. Western blotting analyses showed that GPS significantly inhibited the upregulation of p-Akt, p-MYPT1, and p-MLC protein levels induced by PE in aortic rings without endothelium compared to the PE group (*p* < 0.05), while the levels of total Akt, MYPT1, and MLC were unchanged (Figure 7(a)). In addition, we found that pretreatment with verapamil (L-type Ca^2+^ channel blocker), LY294002 (PI3K/Akt inhibitor), and Y27632 (Rho-kinase inhibitor) significantly attenuated the vasodilation by GPS with *E*_max_ of 28.68 ± 3.43%, 31.52 ± 2.83%, and 39.57 ± 3.39% in aortic rings precontracted with PE, compared to the control group with *E*_max_ of 81.39 ± 3.61% (*p* < 0.05, Figure 7(b)). Thus, these findings confirmed that the vasorelaxation activity of GPS probably involves the PI3K/Akt/Rho-kinase signaling pathway.

## 4. Discussion

Hypertension, angina pectoris, and acute coronary syndrome are usually accompanied by the pathological characters of decreased vasodilation ability and enhanced vasoconstriction that may lead to vessel occlusion and insufficient blood supply to important organs and tissues, which serve as the main major risk factors for cardiovascular diseases [27]. Therefore, improving the vasodilation function of patients is considered an effective method for the prevention and treatment of cardiovascular diseases [28]. To date, rat thoracic aortic rings are used by many researchers as classical models to investigate the vasorelaxation effects of drugs. The present study is the first to investigate the vasodilatory effect of GPS in isolated rat thoracic aorta and to investigate the mechanism of action involved.

Vasodilation can be divided into two types: endothelium-dependent and endothelium-independent. The former is mainly related to the production of endothelium-derived relaxing factor NO and prostaglandins in the thoracic aorta, while the latter is related to a reduction in [Ca^2+^]_in_ levels caused by drugs directly acting on VSM [29]. Contraction of VSM depends on the influx of extracellular Ca^2+^ through VDCC and ROCC in the cell membrane and the release of intracellular Ca^2+^ through stimulation of IP_3_R- and RYR-mediated Ca^2+^ channels in the sarcoplasmic reticulum [30]. KCl-induced contractions mainly result from membrane depolarization and openness of VDCC, while PE, an *α*-adrenoreceptor agonist, leads to an aortic contraction in response to extracellular Ca^2+^ influx through ROCC [31]. Previous studies [32, 33] have demonstrated that 4.2 mM of gentiopicroside exhibited no significant cytotoxicity to chondrocytes, and the methanol extract of *Swertia corymbosa* (family: Gentianaceae) did not produce any mortality and delayed toxicity orally up to 2000 mg/kg (approximately 81.45 mg/kg of GPS) when the animals were monitored for a further 14 days. The data in the present study indicates that GPS dose-dependently produced vasorelaxation effects on aortic rings with endothelium that were precontracted with PE or KCl; this effect of GPS was not significantly different in aortic rings without endothelium. Furthermore, we found that the GPS-induced vasorelaxation effect was unaffected upon pretreatment with NOS inhibitor (L-NAME), COX inhibitor (indomethacin), and sGC inhibitor (methylene blue) in aortic rings with endothelium. These results evidently indicated that the vasodilation effects of GPS on thoracic aortic rings were endothelium-independent, independent of endothelium-derived relaxing factors such as NO and PGI_2_, and that GPS is likely to directly act on the VSM by inhibition of VDCC and ROCC.

K^+^ channels play a critical role in regulating vascular tone by K^+^ efflux, causing cell membrane hyperpolarization, and inhibiting extracellular Ca^2+^ influx leading to vasodilation [34]. The four types of K^+^ channels including K_ATP_, K_ir_, K_Ca_, and K_V_ on the VSM can be blocked using Gli, BaCl_2_, TEA, and 4-AP, respectively. These results showed that the effects of GPS in PE-precontracted rings were attenuated upon treatment with BaCl_2_ and TEA but not 4-AP and Gli, which manifested in the vasodilatory effects related to K^+^ channels. The relationship between Ca^2+^ channels and the vasodilation effects of GPS was further explored in this study. The data showed that GPS dose-dependently attenuated the contraction of high K^+^ depolarized aortic rings induced by gradually increasing CaCl_2_ (0.1-10 mM) input in Ca^2+^-free solution; in addition, GPS obviously decreased the [Ca^2+^]_in_ fluorescence intensity induced by KCl and CaCl_2_ in A7r5 cells, which indicated that GPS inhibited extracellular calcium influx, resulting in vasodilation. PE-induced contractions in Ca^2+^-free solution are ascribed to intracellular Ca^2+^ release via activation of PKC/Rho-kinase- and IP_3_R/RYR-mediated Ca^2+^ sensitization [16, 31, 35]. Our results indicated that GPS treatment significantly decreased Rho-kinase activator- (narciclasine-) and PKC activator- (phorbol-12,13-diacetate-) mediated contraction, and the vasoconstriction of GPS was attenuated upon pretreatment with RYR inhibitor (ruthenium red) and IP_3_R inhibitor (heparin) in aortic rings without Ca^2+^. These results indicated that GPS inhibited the upregulation of [Ca^2+^]_in_ via blockade of both extracellular Ca^2+^ influx in the cell membrane and intracellular Ca^2+^ release through IP_3_R/RYR-mediated Ca^2+^ channels in the sarcoplasmic reticulum by inhibition of VDCC and ROCC, and this inhibitory effect may be closely related to Rho-kinase- and PKC-induced phosphorylation of MLC [11, 36].

**Figure 8:**
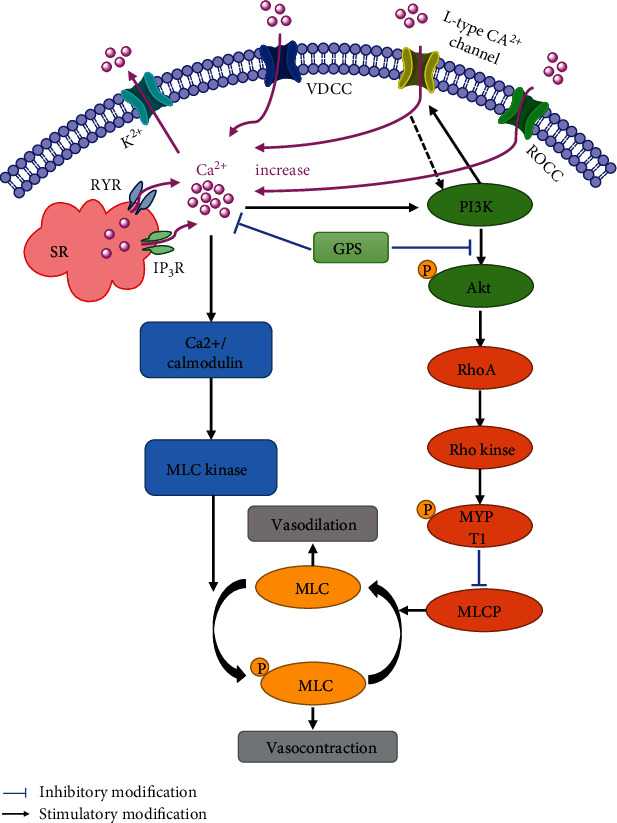
Schematic representation of the underlying mechanisms of the GPS-promoted vasodilatation effects in aortic rings. (1) GPS inhibited extracellular Ca^2+^ influx in the cell membrane and intracellular Ca^2+^ release through IP_3_R- and RYR-mediated Ca^2+^ channels in the sarcoplasmic reticulum by inhibition of VDCC and ROCC. (2) GPS may activate K^+^ channels. (3) GPS inhibited PI3K/Akt/Rho-kinase and L-type Ca^2+^ channel pathways.

It is well documented that activation of the PI3K/Akt pathway significantly enhances the contraction of VSM through stimulation of the L-type Ca^2+^ channel and activation of Rho-kinase [37, 38]. As shown in Figure 8, Ca^2+^ binds to calmodulin in the cytoplasm, activates MLC kinase, phosphorylate MLC, and finally causes vasoconstriction [11, 39]. On the other hand, Rho-kinase can inhibit MLC phosphatase via phosphorylation of MYPT1, resulting in MLC constantly being in the phosphorylated state but not in the dephosphorylated state, which eventually leads to vasoconstriction [40–42]. Our results indicated that treatment with GPS significantly decreased the PE-upregulated levels of Akt, MLC, and MYPT1 phosphorylation. L-type Ca^2+^ channel inhibitor (verapamil), PI3K/Akt inhibitor (LY294002), and Rho-kinase inhibitor (Y27632) were used to confirm that the vasodilatory effect of GPS on aortic rings involves regulation of the PI3K/Akt/Rho-kinase signaling pathway, as evidenced by the fact that vasodilatory effects of GPS were significantly attenuated compared to the control group upon pretreatment with these three inhibitors. Therefore, we suggested that GPS inhibited PI3K/Akt/Rho-kinase signaling pathway leading to vasodilation of aortic rings.

## 5. Conclusions

In summary, the present study demonstrates for the first time that GPS has evident vasodilation effects that are endothelium-independent, and the underlying mechanisms of action for these effects involve the activation of K^+^ channels and inhibition of Ca^2+^ channels by suppressing the activation of the PI3K/Akt/Rho-kinase signaling pathway.

## Data Availability

The research article data used to support the findings of this study are included within the article.

## References

[1] Wang C., Yuan Y., Zheng M. (2020). Association of age of onset of hypertension with cardiovascular diseases and mortality. *Journal of the American College of Cardiology*.

[2] Ning B., Chen Y., Waqar A. B. (2018). Hypertension enhances advanced atherosclerosis and induces cardiac death in Watanabe heritable hyperlipidemic rabbits. *American Journal of Pathology*.

[3] Dunn F. G. (1983). Hypertension and myocardial infarction. *Journal of the American College of Cardiology*.

[4] Rosendorff C. (1997). Endothelin, vascular hypertrophy, and hypertension. *Cardiovascular Drugs and Therapy*.

[5] Hu K., Zhou Q., Jiang Y. (2021). Association between Frailty and Mortality, Falls, and Hospitalization among Patients with Hypertension: A Systematic Review and Meta-Analysis. *Biomed Research International*.

[6] Kearney P. M., Whelton M., Reynolds K., Muntner P., Whelton P. K., He J. (2005). Global burden of hypertension: analysis of worldwide data. *Lancet*.

[7] Bohr D. F., Webb R. C. (1984). Vascular smooth muscle function and its changes in hypertension. *American Journal of Medicine*.

[8] Dong F., Zhang J., Zhu S., Lan T., Yang J., Li L. (2019). Chrysin alleviates chronic hypoxia-induced pulmonary hypertension by reducing intracellular calcium concentration in pulmonary arterial smooth muscle cells. *Journal of Cardiovascular Pharmacology*.

[9] Wilson J. L., Warburton R., Taylor L., Toksoz D., Hill N., Polgar P. (2018). Unraveling endothelin-1 induced hypercontractility of human pulmonary artery smooth muscle cells from patients with pulmonary arterial hypertension. *PloS One*.

[10] Zhu Y., Qu J., He L. (2019). Calcium in vascular smooth muscle cell elasticity and adhesion: novel insights into the mechanism of action. *Frontiers in Physiology*.

[11] Liu Z., Khalil R. A. (2018). Evolving mechanisms of vascular smooth muscle contraction highlight key targets in vascular disease. *Biochemical Pharmacology*.

[12] Niazmand S., Fereidouni E., Mahmoudabady M., Hosseini M. (2017). <i>Teucrium polium</i>-induced vasorelaxation mediated by endothelium-dependent and endothelium-independent mechanisms in isolated rat thoracic aorta. *Pharmacognosy Research*.

[13] Gutierrez A., Contreras C., Sanchez A., Prieto D. (2019). Role of phosphatidylinositol 3-kinase (PI3K), mitogen-activated protein kinase (MAPK), and protein kinase C (PKC) in calcium signaling pathways linked to the *α*1-Adrenoceptor in resistance arteries. *Frontiers in Physiology*.

[14] Miyamoto Y., Feng G. G., Satomi S., Tanaka K., Fujiwara Y., Kinoshita H. (2017). Phosphatidylinositol 3-kinase inhibition induces vasodilator effect of sevoflurane via reduction of Rho kinase activity. *Life Sciences*.

[15] Morello F., Perino A., Hirsch E. (2008). Phosphoinositide 3-kinase signalling in the vascular system. *Cardiovascular Research*.

[16] Kitazawa T., Kitazawa K. (2012). Size-dependent heterogeneity of contractile Ca2+ sensitization in rat arterial smooth muscle. *Journal of Physiology*.

[17] Siobal M. S. (2007). Pulmonary vasodilators. *Respiratory Care*.

[18] Richards J. R., Garber D., Laurin E. G. (2016). Treatment of cocaine cardiovascular toxicity: a systematic review. *Clinical Toxicology*.

[19] Zhou W., Ouyang J., Wang H., Wang X. (2019). Antidermatophyte activity of the Gentiopicroside-rich n-butanol fraction from Gentiana siphonantha Maxim. Root on a Guinea pig model of dermatophytosis. *Complementary Medicine Research*.

[20] Chen C., Wang Y. Y., Wang Y. X. (2018). Gentiopicroside ameliorates bleomycin-induced pulmonary fibrosis in mice via inhibiting inflammatory and fibrotic process. *Biochemical and Biophysical Research Communications*.

[21] Kesavan R., Potunuru U. R., Nastasijević B., T A., Joksić G., Dixit M. (2013). Inhibition of vascular smooth muscle cell proliferation by Gentiana lutea root extracts. *PloS One*.

[22] Rojas A., Bah M., Rojas J. I., Gutiérrez D. M. (2000). Smooth muscle relaxing activity of gentiopicroside isolated from Gentiana spathacea. *Planta Medica*.

[23] Ferro A., Coash M., Yamamoto T., Rob J., Ji Y., Queen L. (2004). Nitric oxide-dependent beta2-adrenergic dilatation of rat aorta is mediated through activation of both protein kinase A and Akt. *British Journal of Pharmacology*.

[24] Huang Y., Wu X., Wu M. (2019). Anti-hypertensive and vasodilatory effects of Qingda granules by suppression of calcium influx and the AKT pathway. *Journal of Cardiovascular Pharmacology*.

[25] Godo S., Shimokawa H. (2017). Endothelial functions. *Arteriosclerosis, Thrombosis, and Vascular Biology*.

[26] Yoshioka K., Sugimoto N., Takuwa N., Takuwa Y. (2007). Essential role for class II phosphoinositide 3-kinase alpha-isoform in Ca2+-induced, Rho- and Rho kinase-dependent regulation of myosin phosphatase and contraction in isolated vascular smooth muscle cells. *Molecular Pharmacology*.

[27] Owen R. S., Carpenter J. P., Baum R. A., Perloff L. J., Cope C. (1992). Magnetic resonance imaging of angiographically occult runoff vessels in peripheral arterial occlusive disease. *New England Journal of Medicine*.

[28] Satoh K. (2017). Development of novel therapies for cardiovascular diseases by clinical application of basic research. *Circulation Journal*.

[29] Yang S., Xu Z., Lin C. (2020). Schisantherin A causes endothelium-dependent and -independent vasorelaxation in isolated rat thoracic aorta. *Life Sciences*.

[30] Marks A. R. (1992). Calcium channels expressed in vascular smooth muscle. *Circulation*.

[31] Hu G. Y., Peng C., Xie X. F., Xiong L., Zhang S. Y., Cao X. Y. (2018). Patchouli alcohol isolated from _Pogostemon cablin_ mediates endothelium- independent vasorelaxation by blockade of Ca^2+^ channels in rat isolated thoracic aorta. *Journal of Ethnopharmacology*.

[32] Zhao L., Ye J., Wu G. T., Peng X. J., Xia P. F., Ren Y. (2015). Gentiopicroside prevents interleukin-1 beta induced inflammation response in rat articular chondrocyte. *Journal of Ethnopharmacology*.

[33] Mahendran G., Thamotharan G., Sengottuvelu S., Bai V. N. (2014). Evaluation of anticonvulsant, sedative, anxiolytic, and phytochemical profile of the methanol extract from the aerial parts of Swertia corymbosa (Griseb.) wight ex C.B. Clarke. *BioMed Research International*.

[34] Dogan M. F., Yildiz O., Arslan S. O., Ulusoy K. G. (2019). Potassium channels in vascular smooth muscle: a pathophysiological and pharmacological perspective. *Fundamental and Clinical Pharmacology*.

[35] Wiciński M., Malinowski B., Rajewski P. (2020). Resveratrol’s impact on vascular smooth muscle cells hyporeactivity: the role of Rho-kinase inhibition. *BioMed Research International*.

[36] Patil S. B., Bitar K. N. (2006). RhoA- and PKC-alpha-mediated phosphorylation of MYPT and its association with HSP27 in colonic smooth muscle cells. *American Journal of Physiology: Gastrointestinal and Liver Physiology*.

[37] Seok Y. M., Azam M. A., Okamoto Y. (2010). Enhanced Ca2+-dependent activation of phosphoinositide 3-kinase class II*α* isoform-Rho axis in blood vessels of spontaneously hypertensive rats. *Hypertension*.

[38] Liu H., Chen Z., Liu J., Liu L., Gao Y., Dou D. (2014). Endothelium-independent hypoxic contraction of porcine coronary arteries may be mediated by activation of phosphoinositide 3-kinase/Akt pathway. *Vascular Pharmacology*.

[39] Raina H., Zacharia J., Li M., Wier W. G. (2009). Activation by Ca2+/calmodulin of an exogenous myosin light chain kinase in mouse arteries. *Journal of Physiology*.

[40] Aburima A., Wraith K. S., Raslan Z., Law R., Magwenzi S., Naseem K. M. (2013). cAMP signaling regulates platelet myosin light chain (MLC) phosphorylation and shape change through targeting the RhoA-Rho kinase-MLC phosphatase signaling pathway. *Blood*.

[41] Szasz T., Webb R. C. (2017). Rho-mancing to sensitize calcium signaling for contraction in the vasculature: role of rho kinase. *Advances in Pharmacology*.

[42] Wilson D. P., Susnjar M., Kiss E., Sutherland C., Walsh M. P. (2005). Thromboxane A2-induced contraction of rat caudal arterial smooth muscle involves activation of Ca2+ entry and Ca2+ sensitization: Rho-associated kinase-mediated phosphorylation of MYPT1 at Thr-855, but not Thr-697. *Biochemicasl Journal*.

